# Heterologous expression of glucose oxidase in the yeast *Kluyveromyces marxianus*

**DOI:** 10.1186/1475-2859-9-4

**Published:** 2010-01-21

**Authors:** Saul N Rocha, José Abrahão-Neto, María E Cerdán, María I González-Siso, Andreas K Gombert

**Affiliations:** 1 Department of Chemical Engineering, Polytechnic School of Engineering, University of São Paulo, CP 61548, 05424-970 São Paulo-SP, Brazil; 2 School of Pharmaceutical Sciences, University of São Paulo, Av Prof Lineu Prestes, 580-bloco 16, 05508-900 São Paulo-SP, Brazil; 3 Departamento de Bioloxía Celular e Molecular, Facultade de Ciencias, Universidade da Coruña, Campus da Zapateira s/n, 15071-A Coruña, Spain

## Abstract

**Background:**

In spite of its advantageous physiological properties for bioprocess applications, the use of the yeast *Kluyveromyces marxianus *as a host for heterologous protein production has been very limited, in constrast to its close relative *Kluyveromyces lactis*. In the present work, the model protein glucose oxidase (GOX) from *Aspergillus niger *was cloned into *K. marxianus *CBS 6556 and into *K. lactis *CBS 2359 using three different expression systems. We aimed at verifying how each expression system would affect protein expression, secretion/localization, post-translational modification, and biochemical properties.

**Results:**

The highest GOX expression levels (1552 units of secreted protein per gram dry cell weight) were achieved using an episomal system, in which the *INU1 *promoter and terminator were used to drive heterologous gene expression, together with the *INU1 *prepro sequence, which was employed to drive secretion of the enzyme. In all cases, GOX was mainly secreted, remaining either in the periplasmic space or in the culture supernatant. Whereas the use of genetic elements from *Saccharomyces cerevisiae *to drive heterologous protein expression led to higher expression levels in *K. lactis *than in *K. marxianus*, the use of *INU1 *genetic elements clearly led to the opposite result. The biochemical characterization of GOX confirmed the correct expression of the protein and showed that *K. marxianus *has a tendency to hyperglycosylate the protein, in a similar way as already observed for other yeasts, although this tendency seems to be smaller than the one of e.g. *K. lactis *and *S. cerevisiae*. Hyperglycosylation of GOX does not seem to affect its affinity for the substrate, nor its activity.

**Conclusions:**

Taken together, our results indicate that *K. marxianus *is indeed a good host for the expression of heterologous proteins, not only for its physiological properties, but also because it correctly secretes and folds these proteins.

## Background

*Kluyveromyces marxianus *is a close relative of *K. lactis*, a model Crabtree-negative yeast, which has been investigated quite extensively by the research community [1-3]. In contrast to the latter, *K. marxianus *has not been the target of systematic investigation efforts. Thus, there is no publicly available genome sequence, no commercial cloning system, and no strain adopted as a reference for basic research purposes for this species [4]. Nevertheless, it has been constantly pointed out as an attractive candidate microorganism for biotechnological applications, due to some of its physiological properties, such as thermotolerance, the capacity of catabolising a broader range of substrates than e.g. *S. cerevisiae*, a strong Crabtree-negative or respiratory metabolism, which leads to high biomass yields on sugar substrates, and high growth rates. In this latter aspect, it has even been labeled the fastest growing eukaryote on the planet [1,5,6].

The lack of an organized research community behind the species *K. marxianus *causes difficulty in extracting objective information about basic aspects of this species. Several different strains have been reported in the literature, many of them not available in the most common international culture collections. On the other hand, the possibilities of exploring this yeast for industrial applications seem to be manyfold. Among them, the production of heterologous proteins is especially attractive, in view of the key physiological properties indicated above. Other yeasts have been more extensively explored for this purpose. Besides *S. cerevisiae*, the most common hosts for the expression of heterologous proteins are *Pichia pastoris*, *Hansenula polymorpha*, *Yarrowia lipolytica*, and also *K. lactis *[3,7-11]. For many of these species, research is quite advanced, with most of the current works focusing on aspects such as the humanization of the secretory pathway, also referred to as glycoengineering, essential for the production of therapeutic proteins, and the search for super-secreting phenotypes [12-14].

Overexpression of foreign genes in *K. marxianus *has been reported in a few articles. After the pioneering work of Bergkamp et al., who expressed alpha-galactosidase of the plant *Cyamopsis tetragonoloba *at 153 mg/L with 99% secretion efficiency (including both the periplasmic and the fully secreted fractions), it took some time before Ball et al. reported on the expression of heterologous beta-glucuronidase or overexpression of homologous beta-glucosidase at levels as high as 10 U/mg protein for the latter enzyme (data obtained from graphs) [15,16]. Later, Almeida et al. expressed the glyceraldehyde-3-phosphate dehydrogenase gene of a flocculent *K. marxianus *strain in another non-flocculent strain of the same species, provoking flocculation in the latter [17]. Pecota et al. reported on the intracellular production of heterologous beta-glucuronidase in *K. marxianus *at ca. 30 U/L (data taken from graphs) [18]. Around the same time, Cai et al. reported on the secretory expression of human interferon alpha-2a at 60 mg/L in a strain of *K. cicerisporus *(a synonym of *K. marxianus*) [19]. Subsequently, Pecota et al. expressed lactate dehydrogenase from *Bacillus megaterium *in *K. marxianus*, which led to the extracellular production of 24 g/L of lactic acid [20]. Hong et al. simultaneously expressed the thermostable endo-beta-1,4-glucanase, cellobiohydrolase, and beta-glucosidase genes, leading to a strain that can grow on cellobiose or carboxymethyl-celulose as sole C-sources [21]. This strain could not produce ethanol from the latter C-source, but presented high ethanol yield on cellobiose. More recently, Nonklang et al. reported on the expression of alpha-amylase from *Aspergillus oryzae *in *K. marxianus*, without providing quantitative data on the levels of heterologous protein obtained [22].

In terms of the glycosylation capacity of *K. marxianus*, there is very limited information available. Siekstele et al. overexpressed the endopolygalacturonase (EPG1) gene of *K. marxianus *in an *EPG1 *deletion strain of the same background [23]. In contrast to native *EPG1 *expression, overexpression of this gene led to an additional band on the protein gel, of higher molecular weight, suggesting that a more glycosylated form of the enzyme is secreted during overexpression, together with the native form. Schwan et al. reported on the native secretion of polygalacturonases in *K. marxianus *[24]. At least four isoenzymes were identified according to the molecular weight, which could be a result of different glycosylation patterns of the same enzyme, according to the authors. Rouwenhorst et al. verified polidispersity of inulinase bands, both when the supernatant and the cell wall (periplasmic) enzyme were analysed, during cultivations of *K. marxianus *CBS 6556 [25]. This observation was attributed to heterogeneity in the size of the polysaccharide chains attached to the inulinase polypeptide.

In this study, the enzyme glucose oxidase (EC 1.1.3.4), which catalyses the oxidation of glucose to gluconolactone and the subsequent reduction of oxygen to hydrogen peroxide was used as model of protein expression in *K. marxianus*. The enzyme contains one very tightly, but nonconvalently bound FAD cofactor per monomer and is a homodimer with molecular mass of 130-320 kDa, depending on the extent of glycosylation. The native enzyme is glycosylated, with a carbohydrate mass percentage of 16-25% [26]. The enzyme expressed in *S. cerevisiae *led to a highly glycosylated form, with a carbohydrate mass percentage approximating 60% [27]. With the aim of evaluating the potential of *K. marxianus *as a host for heterologous protein production, not only in terms of protein levels, but also in terms of glycosylation, localization and thermokinetic properties of the heterologous protein, we cloned the gene for the model protein glucose oxidase from *Aspergillus niger *BT18 into *K. marxianus *CBS 6556 and into *K. lactis *CBS 2359, using different episomal and integrative constructs. This protein offers a great potential for comparison with other expression systems. As far as we are aware, this is the first report of a heterologous protein expressed in *K. marxianus*, for which the glycosylation pattern, localization, stability and kinetic properties were investigated. Three different genetic constructs were used to direct heterologous glucose oxidase expression, which was also followed in the reference yeast *K. lactis*, allowing for a direct comparison between the two organisms.

## Results and Discussion

### GOX expression levels

**Figure 1:**
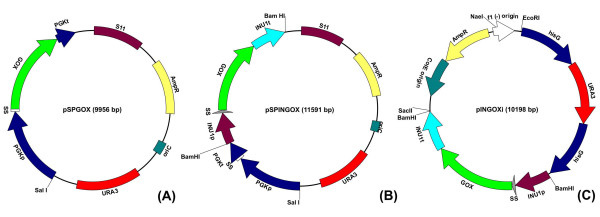
**Expression systems constructed in this work**. (A) pSPGOX provides episomal expression of GOX under control of the *ScPGK *promoter; (B) pSPINGOX is an episomal vector and GOX expression is performed under control of the *KmINU1 *promoter; (C) pINGOXi is an integrative plasmid that confers the cells capacity of GOX expression under control of the *KmINU1 *promoter. (A) and (B) derive from pSPGK1 [48] and (C) corresponds to pNADFL11 [20] plus the expression cassette. Legend: p, promoter; t, terminator; SS, secretion signal sequence.

In the present study, for all constructs (shown on Table 1 and Figure 1), it was possible to detect GOX activity, although in one particular construct (Km1), GOX activity was quite low (Table 2). In all cases, the great majority of the enzyme (> 80%) was secreted from the cells (Table 3), either to the periplasmic space or to the supernatant. The use of *S. cerevisiae *elements for driving GOX expression led to higher heterologous GOX levels in *K. lactis *than in *K. marxianus*. This observation is in accordance with previously reported data by Bergkamp et al., who observed much higher expression of recombinant alpha-galactosidase in *K. lactis *CBS 2359 (90 mg/L), when compared to *K. marxianus *CBS 6556 (2 mg/L), when using either *GLC7 *or *PGK *promoter-driven expression (both promoters from *S. cerevisiae*) [15,28]. Since the level of GOX expression in the Km1 construct was so low, it was not possible to verify whether the secretion signal k1 of *K. lactis *works well for secretion in *K. marxianus*.

**Table 1:** Heterologous GOX constructions.

Construction	Host Organism	Plasmid name	Type	Promoter	Signal sequence	Terminator
Km1	*K. marxianus*	pSPGOX	Episomal	*Sc PGK*	*Kl *k1	*Sc PGK*
Km2	*K. marxianus*	pSPINGOX	Episomal	*Km INU1*	*Km INU1*	*Km INU1*
Km3	*K. marxianus*	pINGOXi	Integrative	*Km INU1*	*Km INU1*	*Km INU1*
Kl1	*K. lactis*	pSPGOX	Episomal	*Sc PGK*	*Kl *k1	*Sc PGK*
Kl2	*K. lactis*	pSPINGOX	Episomal	*Km INU1*	*Km INU1*	*Km INU1*
Kl3	*K. lactis*	pINGOXi	Integrative	*Km INU1*	*Km INU1*	*Km INU1*

List of the six constructions obtained in this work and their details.

**Table 2:** GOX enzymatic activities.

**Construction**^**(a)**^	**Final Biomass**^**b**^	**GOX activity**^**(c)**^						
		
		Supernatant		Cell wall		Cell-bound		Total
	
	(gDW/L)	U/mgProt	U/gDW	U/mgProt	U/gDW	U/mg Prot	U/gDW	U/gDW
Km1	7.4 ± 0.3	0.3 ± 0.1	0.5 ± 0.1	2.4 ± 0.1	1.9 ± 0.1	0.03 ± 0.02	4.0 ± 1.4	7.4 ± 2.5
Km2	12.1 ± 0.2	251 ± 72	407 ± 16	180 ± 24	1145 ± 35	1.3 ± 0.2	171 ± 44	1722 ± 62
Km3	12.0 ± 0.1	171 ± 44	255 ± 1	187 ± 8	850 ± 70	2.4 ± 0.3	291 ± 27	1395 ± 42
Kl1	9.9 ± 0.2	40 ± 3	161 ± 17	16 ± 1	13 ± 1	0.3 ± 0.1	31 ± 2	205 ± 16
Kl2	11.2 ± 0.0	32 ± 1	94 ± 6	64 ± 5	49 ± 8	0.1 ± 0.0	16 ± 3	158 ± 17
Kl3	9.6 ± 0.2	29 ± 1	94 ± 12	76 ± 4	64 ± 0	0.1 ± 0.0	13 ± 1	170 ± 11

Heterologous GOX production in the supernatant, cell wall and cell-bound fractions of different *K. marxianus *and *K. lactis *strains.

(a) see Materials and Methods section, Table 1 and Figure 1 for a description of the genetic constructions.

(b) see Materials and Methods section, for a description of the cultivation conditions.

(c) subcellular distribution according to Rouwenhorst et al. [50]

**Table 3:** GOX distribution.

**Construction**^**(a)**^	**GOX activity per g DW in each fraction relative to the total**^**(b, c)**^		
	
	Supernatant	Cell wall	Cell-bound
Km1	9% ± 3%	31% ± 8%	62% ± 11%
Km2	23% ± 2%	67% ± 1%	10% ± 3%
Km3	19% ± 1%	61% ± 3%	21% ± 2%
Kl1	79% ± 2%	6% ± 0%	16% ± 2%
Kl2	60% ± 2%	31% ± 2%	10% ± 0%
Kl3	50% ± 3%	38% ± 3%	10% ± 3%

Subcellular distribution of heterologous GOX in different *K. marxianus *and *K. lactis *strains.

(a) see Materials and Methods section, Table 1 and Figure 1 for a description of the genetic constructions.

(b) see Materials and Methods section, for a description of the cultivation conditions.

(c) subcellular distribution according to Rouwenhorst et al. [50]

A strong increase in the levels of heterologous GOX was obtained in *K. marxianus *when the homologous *INU1 *promoter and secretion signal were employed to drive heterologous gene expression, when compared to the use of a *S. cerevisiae *promoter with a *K. lactis *secretion signal. This also agrees with the results reported by Bergkamp et al., who obtained 153 mg/L of heterologous alpha-galactosidase (including both the periplasmic and the supernatant fractions) in *K. marxianus*, using the *INU1 *promoter and secretion signal, compared to 2 mg/L when the *S. cerevisiae *elements mentioned above were employed [15]. These authors estimated the plasmid copy number per cell to be 25 in their studies. However, this much higher expression level of alpha-galactosidase obtained using the *INU1*-derived constructions cannot be explained exclusively by the increased plasmid copy number (the expression level increased 75 times), and is probably also caused by an increased plasmid stability and promoter strength in the host. Interestingly, the amount of heterologous protein obtained in the present work is very similar to the one obtained in the referred work. If we assume a conversion factor of 80-172 U/mg protein for glucose oxidase, we can estimate the extracellular production of glucose oxidase in the Km2 construct to be between 90 and 194 mg/L [29]. Bergkamp et al. used a conversion factor of 100 U/mg to calculate the 153 mg/L of heterologous protein obtained in their work [15,28].

The enhancing effect of the *INU1 *promoter and secretion signal on GOX expression was exclusive to *K. marxianus*, since in the *K. lactis *constructs using this system (Kl2 and Kl3), the levels of total GOX produced were even slightly lower than the levels attained with the *S. cerevisiae *promoter and k1 secretion signal (Kl1) (Table 2). In particular, the secretion efficiency of heterologous GOX using the *KmINU1 *pre-pro sequence was much lower in *K. lactis *than that obtained when the homologous k1 signal was employed.

When both *INU1*-based expression systems are compared in *K. marxianus*, it can be seen that the amounts of GOX produced were around the same level, slightly higher in the episomal construct (Km2) than in the integrative one (Km3) (Table 2).

Considering that integration occurs preferably at a single copy in the genome and that replicative plasmids multiply autonomously in the cytoplasm, the similar GOX expression levels achieved in the two systems mentioned above are probably due to a combination of a higher copy number of pSPINGOX at the beginning of the cultivation and a concomitant loss of plasmids during the cultivation, when compared to the stable Km3 transformant (integrative). According to plasmid stability studies (data not shown), after 48 h of cultivation, both cells of *K. marxianus *and *K. lactis *nearly lost their episomal pSPGK1-derived plasmids.

Additional file 1 (Table S1) shows an overview of heterologous expression of *Aspergillus niger *GOX in different microorganisms. A direct comparison among the different expression systems is difficult, due to the different cultivation strategies used in each case. Taking this into account, the levels of GOX activity achieved in the present work with *K. marxianus *are in a low-to-middle range, if we consider the highest (30,000 U/g DW, assuming a final biomass concentration of 15 g/L) and the lowest (27.9 U/g DW) values published (Additional file 1 - Table S1) [30,31]. However, since the aim of the present work was not to optimize the levels of heterologous GOX expression, there is probably much room for increasing these levels in further studies.

### Biochemical characterization of heterologous GOX

**Figure 2:**
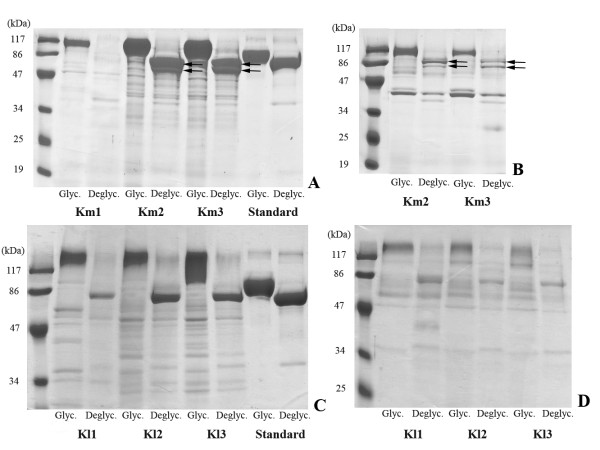
**Coomassie blue stained SDS-PAGE for the analysis of the glycosylation pattern of GOX expressed in (A, B) *K. marxianus *and (C, D) *K. lactis***. (A) *K. marxianus *cell wall-associated GOX; (B) *K. marxianus *supernatant GOX; (C) *K. lactis *cell wall-associated GOX; (D) *K. lactis *supernatant GOX. Glyc: glycosylated enzyme. Deglyc: deglycosylated enzyme with PNGase F, as described in Methods. Construction codes are listed in Table 1. Standard: commercial GOX from *A. niger *(Product B6916, Sigma, USA), solubilized in water (Glyc) or deglycosylated (Deglyc) as described above. The arrows indicate the two bands resulting from the deglycosylation of GOX expressed in *K. marxianus*.

**Figure 3:**
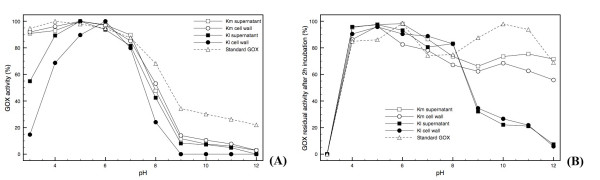
**Effect of pH on heterologous GOX activity**. (A) GOX activity was measured from pH 3 to 12; and (B) residual GOX activity was measured after 2 hours of enzyme incubation at the corresponding pH (here, activity measurements were always carried out at 37°C and pH 4.5). The following buffers were used: acetate (3.0, 4.0, 5.0); HEPES (6.0, 7.0, 8.0); Tris (9.0, 10.0, 11.0, 12.0). Each point is the mean of a duplicate assay. Km supernatant and Km cell wall: GOX produced by Km2 construction. Kl supernatant and Kl cell wall: GOX produced by Kl1 construction. Both clones were cultivated as described in the Methods section. Standard: commercial GOX from *A. niger *(Product B6916, Sigma, USA).

**Figure 4:**
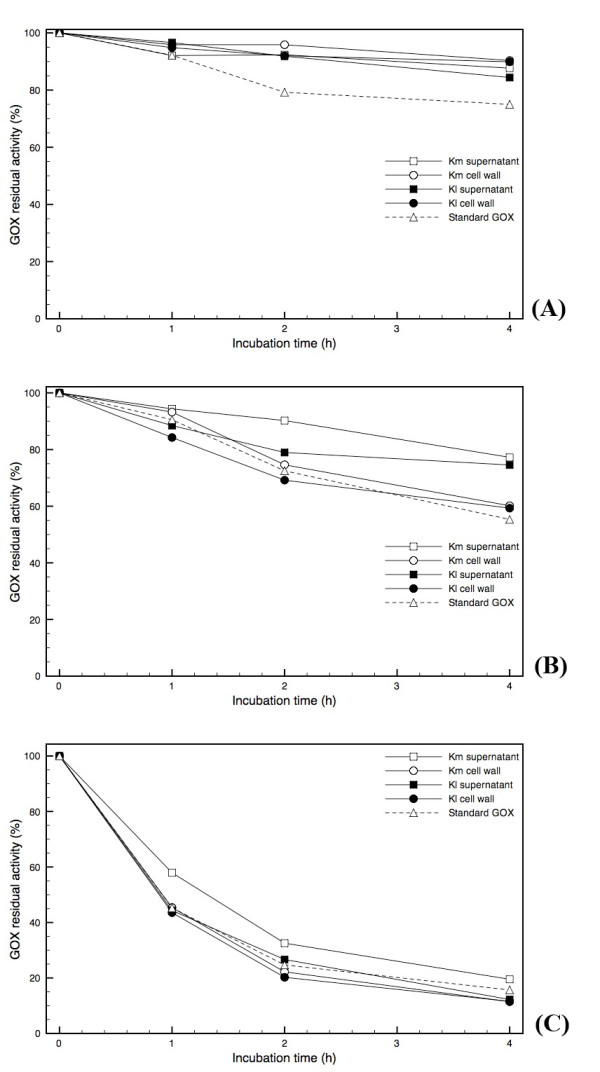
**GOX stability at different temperatures**. Time course of residual GOX activity was measured after incubation at (A) 37°C; (B) 55°C, and (C) 60°C. Activity measurements were always carried out at 37°C and pH 4.5. Each point is the mean of a duplicate assay. The error bars show standard deviations. Km supernatant and Km cell wall: GOX produced by Km2 construction. Kl supernatant and Kl cell wall: GOX produced by Kl1 construction. Both clones were cultivated as described in Methods section. Standard: commercial GOX from *A. niger *(Product B6916, Sigma, USA).

The biochemical characterization of GOX performed here, in terms of molecular weight estimation using denaturing gels (Figure 2), activity under different pH values (Figure 3), stability towards pH (Figure 3), stability towards temperature (Figure 4), and in terms of the apparent Michaelis-Menten constant (Table 4) confirm that both yeasts were able to express and secrete the expected heterologous protein.

The pH for maximal activity lies between 5 and 6 for all cases investigated (Figure 3). The GOX activity-versus-pH profile, shown by the enzyme produced in *K. marxianus*, was closer to the standard enzyme in the range between the values of 3 and 7, when compared to the GOX produced by *K. lactis*. An optimum pH value of 5.5 was obtained by Zia et al. [32]. In alkaline pH values, the standard GOX is slightly more active than the ones expressed in either *K. marxianus *or *K. lactis*. The GOX secreted from *K. marxianus *has its structure partially affected at alkaline pH values, which becomes evident by its decreased activity, similarly to what occurs with the standard enzyme (Figure 3), since after 2 hours of exposure to these conditions, the activity of both enzymes could be restored by assaying the enzyme at the optimal pH (Figure 3B). The enzyme released from *K. lactis *is different, since its original activity was not fully restored under such conditions (Figure 3B). Thus, hyperglycosylation, an event that occurred to a higher extent in K. lactis than in *K. marxianus *(Figure 2), seems to provoke a conformational change that makes the protein irreversibly loose catalytic activity, especially during exposure to alkaline pH values (Figure 3B). Nevertheless, it is possible to affirm that, in general, the behavior of both heterologous enzymes is very similar to that of the standard GOX, mainly between pH 4 and 8.

**Table 4:** K_M(app) _values with respect to glucose.

GOX origin	K_M (app)_
Km2 supernatant	22.8 mM
Km2 cell wall	17.4 mM
Kl1 supernatant	24.4 mM
Kl1 cell wall	22.6 mM

Standard GOX	16.4 mM

List of GOX K_M (app) _determined from different constructions and fractions using the Lineweaver-Burk linearization method. K_M (app) _was determined as described in the Methods section.

In terms of stability towards temperature, all enzymes presented a similar behavior. All enzymes remain quite stable after 4 hours of incubation at 37°C. At a temperature value above 55°C, but below 60°C, the stability of the enzyme starts to be strongly affected (Figure 4). A very similar thermal inactivation pattern was obtained by Gouda *et al*., who observed a significant loss of *A. niger *GOX activity at temperatures above 50°C [33].

The apparent Michaelis-Menten constants are all very similar, approaching the value obtained with the standard GOX (Table 4), which can be considered quite pure from the gels on Figure 2. There is not a consensus in literature concerning GOX K_M _values. Wong et al. [29] reviewed several authors who published values ranging from 33 mM (at 25°C) to 248 mM (at 30°C). Zia et al. published a K_M _of 2.56 mM (at 40°C) for a GOX produced by *A. niger *UAF-1 [32]. The same authors obtained a K_M _of 28 mM for the enzyme produced by *A. niger *BCG-5, a gamma radiation-mutant strain [34]. Mirón et al. estimated a value of 18 mM (at 30°C) [35]. Kohen et al. [26] calculated K_M _to be 28 mM at 25°C. This large dispersion of values is probably a consequence of the different conditions employed in the activity assays.

In Figure 2 it is possible to see that, after removal of the carbohydrate moiety of heterologous GOX with PNGase F, two bands appear on the gel corresponding to *K. marxianus *(indicated by the arrows), whereas only one band is visible on the gel corresponding to *K. lactis*. Since only one band is also visible after deglycosylation of the standard GOX with the same deglycosylating enzyme, this indicates that the glycosylation patterns in *K. marxianus *and *K. lactis *are different. It could be that in *K. marxianus *O-glycosylation occurs, besides N-glycosylation and that this is not the case for *K. lactis *[36,37]. Another observation is that the glycosylated GOX in *K. lactis *has a higher molecular weight than the one in *K. marxianus*, indicating that the latter yeast has a lower tendency to hyperglycosylate proteins than the former, and consequentely also than *S. cerevisiae *[38]. Bergkamp *et al*. also observed a slightly higher molecular weight of the same alpha-galactosidase expressed in *S. cerevisiae *in comparison to the one produced by *K. marxianus*, which is most probably due to a higher glycosylation pattern in the former yeast [15]. Additional file 1 (Table S1) shows that GOX glycosylation levels in *K. marxianus *are close to the ones obtained in *H. polymorpha*, which are the lowest values obtained for heterologous GOX expression in yeasts [39,40]. It can also be seen from the gels (Figure 2) that the periplasmic and the fully secreted GOX have the same or at least a very similar molecular weight in both yeasts.

The bands corresponding to GOX in *K. lactis *are less sharp than the ones in *K. marxianus*. This could be due to some degradation of the enzyme during its extraction from the periplasmic space. When the lanes corresponding to the supernatant (Figure 2D) are compared to the lanes corresponding to the sample after extraction from the periplasmic space (Figure 2C), it can be seen that there is much less non-specific protein in the former case, when compared to the latter. This is probably due to a general protein release from the periplasm during the extraction procedure.

The heterologous protein glycosylation pattern can be an important parameter when designing a commercial producing system. In the particular case of GOX, Kohen et al. [26] discuss that glycosylation does not affect the protein catalytic parameters (substrate binding and product releasing) when glucose is used as substrate. Moreover, the authors affirm that there is no evidence that glycosylation causes a major change in protein structure.

The notion that *K. marxianus *has a smaller tendency to hyperglycosylate heterologous proteins, when compared to other yeasts, needs to be confirmed by expressing other proteins in this organism and, in case it is confirmed, it would contribute to making this host attractive for the expression of therapeutic proteins.

## Conclusions

We used the model protein glucose oxidase (GOX) from *Aspergillus niger *to evaluate the potential of the yeast *K. marxianus *to be used as a host for the production of heterologous proteins and to compare this system to the various heterologous GOX expression systems described in other organisms. Since GOX has eight potential sites for *N*-glycosylation, this enzyme also becomes suitable for a comparison of glycosylation levels in *K. marxianus *and in *K. lactis*. Besides this, it allows for an evaluation of how different glycosylation levels influence enzyme performance, mainly its activity and other biochemical properties, such as stability. For this purpose, we employed different episomal and integrative constructs, containing genetic elements either from *S. cerevisiae *in combination with elements from *K. lactis *or from *K. marxianus*. These expression systems were transformed into *K. marxianus *and *K. lactis*, in order to allow for a direct comparison of GOX expression in both yeasts. The biochemical characterization of the secreted enzyme, in terms of its localization, glycosylation pattern, pH range of activity, stability towards different temperatures and pH values, and apparent Michaelis constant, showed that GOX was expressed and secreted in active form in both species. The highest values of secreted GOX achieved were 1552 U/g dry cell weight of *K. marxianus *CBS 6556, using an episomal expression system with a promoter, prepro sequence and terminator of the homologous *INU1 *gene. GOX expression based on *INU1 *genetic elements yielded higher levels in *K. marxianus *than in *K. lactis*. On the other hand, GOX expression based on the *ScPGK *promoter and terminator, in combination with the killer k1 secretion signal from *K. lactis*, led to higher levels in the latter yeast than in *K. marxianus*. *K. lactis *showed a higher capacity of GOX full secretion (in relative terms), when compared to *K. marxianus*, which retained more of the enzyme in the periplasm. In terms of glycosylation, it seems that the heterologous GOX expressed in *K. marxianus *was less hyperglycosylated than the one expressed in *K. lactis *and that hyperglycosylation did not negatively affect enzyme activity or stability. In conclusion, this paper provides data indicating that *K. marxianus*, which is a faster growing organism, more tolerant towards high temperatures and presenting a broader substrate range than its relative *K. lactis*, seems to be a suitable host for the production of glycosylated heterologous proteins.

## Methods

### Strains and media

*Escherichia coli *strain DH10b (Invitrogen, USA) was used for plasmids construction and propagation. *Kluveromyces marxianus *CBS 6556 was purchased from Centraal Bureau voor Schimmelcultures (Ultrecht, The Netherlands). *Kluveromyces lactis *PM5-3C (*MATa uraA Rag*^+^) was kindly provided by Dr. Micheline Wesolowski-Louvel (Lyon, France). *Saccharomyces cerevisiae *BY4742 (*MATα his3D1 leu2D0 lys2D0 ura3D0*) was provided by Euroscarf (Frankfurt, Germany) [41]. The strain *K. marxianus *SLC33 is an *ura3 *mutant from *K. marxianus *CBS 6556, obtained in this work by selection in 5-FOA-containing plates (*ura*- phenotype stability was certified for up to 120 generations).

*E. coli *strains were grown on LB (1% bacto-tryptone, 0.5% yeast extract, 0.5% NaCl, 0.1% glucose) liquid or solid (2% bacto-agar) media at 37°C. Ampicilin (40 mg/L final concentration) was added for plasmid selection. For the selection of yeast transformants exhibiting *URA3 *expression, complete medium (CM) lacking uracyl (CM-URA) was used [42]. 5-FOA plates for selection of *ura3 *mutants were made as described in [43]. A first screening of GOX-expressing yeasts was performed in YPHSM-SUC medium, which contains (in g l^-1^): peptone (80), yeast extract (10), glycerol (30), sucrose (10). For the GOX expression studies, strains were grown on the defined medium (DM) described by [44], and subsequently on YPS complex medium (1% yeast extract, 2% bacto-peptone, 2% sucrose).

### Isolation and manipulation of nucleic acids

Plasmid DNA from *E. coli *was obtained using the Spin Clean Plasmid Miniprep Kit (Mbiotech, South Corea). Plasmid DNA from yeast cells was prepared using the same kit of bacterial mini-prep, differing in some steps of the protocol, as follows: yeast cells containing the plasmid of interest were harvested from a 24-hour, 10 mL culture on CM medium, by centrifugation (3000 × g, 5 min). The pellet was resuspended in 250 μL of the resuspension buffer and 250 μL of the lysis buffer (provided with the kit) and approximately 250 μL of glass beads (425-600 μm, Sigma, USA). The mixture was vortexed vigorously for 2 minutes and maintained in ice for other 5 minutes. Afterward, the ordinary mini-prep protocol was followed. The final DNA suspension was used directly for *E. coli *transformation and plasmid propagation. Yeast transformation by the lithium acetate method was performed as described by [45]. Furthermore, yeast transformation by electroporation was adapted from the protocol described by [46], as follows. 10 mL of a yeast culture in early exponential phase were centrifuged (4000 × g, 5 min) and washed with 1 mL of ultrapure water. Subsequently, cells were resuspended in 1 mL of pre-treatment buffer (YPD, 200 mM DTT, 300 mM HEPES) and incubated at 30°C for 30 minutes. Then, cells were centrifuged (4000 × g, 5 min) and resuspended in 1 mL of electroporation buffer (10 mM Tris-HCl pH 7.5, 270 mM sucrose, 1 mM lithium acetate). The electric shock was carried out using the following parameters: 25 μF, 550 V, and 600 Ω.

PCR reactions were carried out in a final volume of 25 μL of reaction mixture which consisted of 20 ng of DNA template, 30 pmol of each primer (Table 5), 1.75 nmol of each dNTPs, 50 mM KCl, 2.5 mM MgCl_2_, 0.25 U of Taq DNA polymerase (Roche Diagnostistics, Mannheim, Germany) in 10 mM Tris-HCl buffer, pH 8.3. The reactions were proceeded in 30 cycles: denaturation was at 94°C for 30 seconds, annealing at 50°C (for the GOX gene) and at 53°C (for the *INU1 *cassette) for 60 seconds, and extension at 72°C for 2.5 minutes (GOX) and 3 minutes (*INU1*). All other DNA manipulations were performed as described by [47].

**Table 5:** Primers.

Primer ID	Sequence	Details
PSPGOXF	5' - TTTTTGCTGTCATTCGTTCAAGGTAAAAGAAGCAATGGCATTGAAGCC	Underlined sequence is homologous to the last bases of *K. lactis *killer protein k1 secretion signal sequence
PSPGOXR	5' - CACCACCACCAGTAGAGACATGGGAGATCGTCACTGCATGGAAGCATA	Underlined sequence is homologous to the first bases of *S. cerevisiae *PGK terminator
PINU1Fb	5'- GGATCCGAATTCTCAAACCGAAATGGG	Underlined extremity is an added *Bam*HI restriction site
PINU1Rb	5'- GGATCCACGCCAGACGTCTTGTGTCCG	Underlined extremity is an added *Bam*HI restriction site
PINUGOXF	5' -GTCAGTGCTTCAGTTATCAATTACAAGAGA AGCAATGGCATTGAAGCC	Underlined sequence is homologous to the last bases of *K. marxianus INU1 *secretion signal sequence
PINUGOXR	5'- TTTTGTCGTTAGTAAAGTAAGCAGATCAGA TCACTGCATGGAAGCATA	Underlined sequence is homologous to the last bases of *K. marxianus INU1 *terminator

List of primers used on DNA constructions.

### Plasmids construction and yeast transformation

The episomal expression vector pSPGOX (Fig. 1A) was constructed as follows. The GOX gene of *A. niger *BT18 was amplified by PCR from the plasmid pBT86 (obtained from Dr. Oscar Bañuelos, Puleva Biotech Inc., Spain) using the primers PSPGOXF and PSPGOXR (Table 5). The amplified 1.8 kb fragment was cloned into the replicative plasmid pSPGK1 [48]. This plasmid possesses the *K. lactis *S11 origin of replication and owns the ability to replicate in *K. marxianus *and *K. lactis *cells. The insertion of GOX CDS was performed by homologous recombination of the PCR product with pSPGK1 linearized with *EcoRI*. Equal amounts of plasmid and insert (500 ng) were used to transform *K. lactis *PM5-3C by the lithium acetate method. Transformants showed ability to grow on CM-URA selective medium. Confirmation of recombination was perfomed by PCR, amplifying the GOX fragment from the plasmids with PSPGOXF and PSPGOXR and by the extracellular expression of glucose oxidase by top-agar plate assay described by [49]. Hence, plasmids were extracted from *K. lactis *cells and used to transform *K. marxianus *SLC33 by the lithium acetate method.

The constructions using *INU1 *cassette for GOX expression started with amplification of the *INU1 *cassette from the chromosomal DNA of *K. marxianus *CBS 6556 using primers PINU1Fb and PINU1Rb (Table 5). Both primers have in the 5' extremities a *Bam*HI restriction site. After amplification, the *INU1 *cassette was ligated to the pMBL1-T (MBiotech, South Korea) vector for propagation. Thus, the insert was excised by *Bam*HI restriction. Later, this cassette was cloned into the yeast-*E. coli *shuttle vector Yeplac195, and the resulting construct linearized with *Tth*111I. In parallel, GOX from pBT86 was amplified using primers PINUGOXF and PINUGOXR (Table 5). These two primers were capable to add extremities to the amplified gene, which were homologous to *INU1 *secretion signal and terminator sequences. The resulting fragment was cloned into pMBL1-T for propagation. For recombination, the linearized Yeplac195 and the amplified GOX carrying homologous extremities were fused by homologous recombination in *S. cerevisiae *BY4742 transformed with an equal proportion of both plasmid and insert (500 ng) by the lithium acetate method. The construction was purified from *S. cerevisiae *cells and digested with *Bam*HI, which released the GOX expression cassette. After propagation of the 3.4 kb cassette in pMBL1-T, this fragment was subcloned into the *Bam*HI site of pSPGK1 and pNADFL11, resulting in the episomal and integrative constructions shown in Figures 1B and 1C, respectively.

The expression vectors were used to transform *K. marxianus *SLC33 and *K. lactis *PM5-3C by the lithium acetate method, with exception of the integrative construction in *K. marxianus*. In this case, the yeast was transformed by electroporation, since the lithium acetate method was ineffective. The resulting constructs are detailed on Table 1 and Figure 1.

After transformation of yeast cells with each construction, transformants that showed higher extracellular GOX activity were selected by top-agar. Colonies exhibiting higher activities were surrounded by a larger brownish halo. The three transformants showing higher activities of each construct were selected. Thus, the chosen candidates were grown in liquid YPHSM-SUC medium for 36 h and GOX activity measured. The candidates that exhibited the higher activity for each construct were selected for the further studies.

### Analysis of glucose oxidase production in submerged cultivations

The cultivation conditions described by [15], which resulted in the efficient secretory production of a heterologous alpha-galactosidase in *K. marxianus*, were adopted with slight modifications. Transformants were grown in baffled 500 mL shake flasks closed with cotton on an orbital shaker (300 rpm), using 100 mL of medium at 30°C. First, the cells were grown for 24 h in the defined medium DM (using 2% glucose as carbon source). Second, these cells were diluted 1:10 into fresh YPS medium and grown for another 48 h. All cultivations were performed in duplicate. Subsequently, the heterologous enzyme present in the supernatant, in the cell wall (retained inside the periplasmic space), and the cell-bound (intracellular) fractions were separated based on the method described by Rouwenhorst et al. [50]. The solubilization of cell wall-associated enzyme was induced by suspension of the cells in 10 mL of enzyme release buffer (50 mM potassium phosphate, pH 7, 10 mM 2-mercaptoethanol, 10 mM dithiotheitol, 2 mM MgSO_4_). The cell-bound enzyme was liberated incubating cells in 100 mM phosphate buffer containing 2 mM MgCl_2_, 2% DTT and 5% protease inhibitor cocktail (Product no. P8215, Sigma, USA). Glass beads (425-600 m) were added, and total cell disruption was performed by vortexing the mixture five times for 1 minute (with 1 min interval, incubated in ice). Cell debris was separated by centrifugation and the supernatant contained the cell-bound enzyme. GOX activity was determined spectrophotometrically in the three fractions, relative to a standard curve of absorbance vs enzyme activity, by the o-dianisidine reduction method [49]. Protein content in all the fractions was measured by the Bradford method (product B6916, Sigma, USA), with bovine serum albumin as standard, according to the instructions of the manufacturer. The dry cell weight of 5 mL sample of each culture flask was determined using 0.45 μm membrane filters and a microwave oven (180 W, 15 min) [51].

### GOX glycosylation analysis

The glucose oxidase expressed by the six different constructions of *K. marxianus *and *K. lactis *(Table 1) was analyzed with respect to the degree of glycosylation in the supernatant and in the cell wall fractions (the cell-bound fraction was always very small, compared to the two other fractions). For this purpose, the supernatant and cell wall fractions were concentrated in 10 kDa cut-off ultrafilters (Millipore, USA). To de-glycosylate GOX, 15 μg of the protein were denaturated by incubation for 5 min at 100°C in 10 μL final volume reaction containing 0.5% SDS and 40 mM 2-mercaptoethanol. After cooling, Tergitol NP-40, sodium phosphate buffer pH 7.5, and PNGase F (New England Biolabs, USA) were added to final concentrations of 1%, 50 mM, and 5 U/μL, in a 20 μL final volume reaction. The mixture was incubated for 1 hour at 37°C. Migration patterns of both glycosylated and deglycosylated forms of GOX were compared on a 10% SDS-PAGE gel carried out as described by Laemmli [52] and stained with coomassie blue.

### GOX biochemical characterization

The enzyme synthesized by the transformant of each species, either *K. marxianus *or *K. lactis*, that exhibited the highest biomass specific activity in both fractions (supernatant and cell wall) was subjected to a biochemical characterization, in which the pH of optimal activity and the stability at different pH and temperature values were examined. GOX activity was measured at the following pH values: 3.0, 4.0, 5.0 (50 mM acetate buffer); 6.0, 7.0, and 8.0 (50 mM HEPES buffer); 9.0, 10.0, 11.0, 12.0 (50 mM Tris buffer). In each buffer, glucose was mixed to a final concentration of 100 mM, 2 U horseradish peroxidase (Type II, Sigma, USA), and o-dianisidine di-hydrochloride to a final concentration of 100 μM. The thermal stability was determined during four hours of incubation at 37°C, 55°C, and 60°C at pH 4.5. Samples were taken at 0, 1, 2 and 4 hours of incubation and the enzyme activity was measured immediately at 37°C and pH 4.5. Stability towards pH was determined after incubating the enzyme for 2 hours at 37°C in the buffers mentioned above (from pH 3.0 to 12.0). Samples were immediately assayed for enzyme activity at pH 4.5. For the K_M (app) _determination, the supernatant and cell wall fractions were purified in a Sephacryl S-300-HR gel-permeation column, in order to eliminate reactive contaminants. Samples were eluted at 0.5 mL/min in 50 mM citrate buffer (pH 4.8). K_M (app) _was determined measuring the lambda absorbance per minute increase at 30°C, incubating the enzyme with 50 mM McIlvane's buffer, 2 U horseradish peroxidase (Type II, Sigma, USA), and 100 μM o-dianisidine di-hydrochloride, and glucose in the following concentrations: 2 mM, 5 mM, 10 mM, 15 mM, 20 mM, 30 mM, 50 mM, 75 mM, 100 mM. All biochemical assays were also performed with a commercial *A. niger *GOX (G6125, Sigma, USA), considered here as a standard.

## Competing interests

The authors declare that they have no competing interests.

## Authors' contributions

SNR participated in the design of this study, carried out the practical experiments, data analysis and drafted part of the manuscript. JA-N participated in designing and executing the GOX biochemical characterization and glycosylation studies, in the analysis of results, reviewing and commenting the manuscript. MEC participated in reviewing and commenting the manuscript. MIG-S designed and supervised all DNA construction strategies, and participated in the analysis of results, writing, reviewing and commenting the manuscript. AKG conceived the study, designed the cultivations, participated in the analysis of results, writing, reviewing and commenting the manuscript. All authors have read and approved the manuscript.

## Supplementary Material

Additional file 1 **Table S1 - Different systems for GOX expression**. Overview of different microbial systems employed for the heterologous expression of *Aspergillus niger *glucose oxidase. Click here for file
