# Supplementary material for: Heterologous expression of glucose oxidase in the yeast Kluyveromyces marxianus
Source: Microb Cell Fact. 2010 Jan 21;9:4. doi: 10.1186/1475-2859-9-4 (PMC2817671; doi:10.1186/1475-2859-9-4)
Supplement: Additional file 1 — Table S1 - Different systems for GOX expression. Overview of different microbial systems employed for the heterologous expression of Aspergillus niger glucose oxidase. [file 1475-2859-9-4-S1.DOC]

| **Micro-organism** | **Expression system** | **Promoter** | **Secretion signal** | **Extracellular level^(a)^** | **Medium** | **Cultiva-tion in** | **Process optimized?** | **Glycosylation study** | **Ref** |
| --- | --- | --- | --- | --- | --- | --- | --- | --- | --- |
| *Hansenula polymorpha* | Episomal | *MOX1* | *A. oryzae* α-amylase | NP | Complete medium (lacking leucine) + 2% glucose (12h, preculture); Complex + 2% methanol (36h, cultivation) | Shake-flask | No | GOX from *H. polymorpha* (200 kDa) was less glycosylated than the one expressed in *S. cerevisiae* (220 kDa), but more than the one from *A. niger* (160 kDa). | [38] |
| *Hansenula polymorpha* | Episomal | *MOX1* | *A. oryzae* α-amylase | NP | Complex + 2% glucose (12h, preculture); Complex medium + 2% methanol (36h, cultivation) | Shake-flask | No | GOX from *H. polymorpha* was less glycosylated when cultivated in medium containing sorbitol (approx. 190 kDa). | [39] |
| *Mucor circinelloides* | Episomal | A. nidulans *gpd1* | *A. niger* gox | Present, but not quantified. | Complex + 2% glucose (cultivation) | Biore-actor | No | No. | [40] |
| *A. niger* | Episomal | A. nidulans *gpd1* | *A. oryzae* α-amylase | Maximum level: 27.9 U/gDW | Minimal medium + 0.2% yeast extract + glucose 8% (60h, cultivation) | Biore-actor | Yes. From shake flasks to bioreactor. There was also opmization in medium composition. | No. | [31] |
| *S. cerevisiae* | Episomal | *GAL1, GAL10,*  *GPD, AG (ADH2* UAS:: *GPD* TATA) | Part of *A. niger* gox (40 bp upstream the translational start codon) | Maximum level: 460 U/mL. (with an UV mutant, under control of AG promoter). | Complete medium (lacking uracyl + 2% glucose (24h, preculture); complex medium + 3% corn steep liquor (72h, cultivation) | Shake- flask | Yes. Glucose and corn steep liquor were tested. Kinetic studies of GOX production were performed in bioreactor. UV mutagenesis was used to increase GOX production. | There is a mention that the GOX expressed in *S. cerevisiae* subunit show a typical broad band around 110 kDa in polyacrylamide gel electrophoresis. | [30] |
| *S. cerevisiae* | Episomal and integrative | *PGK1* | *S. cerevisiae MFα* | Maximum attained level (approx. 90 U/mL). | Complete medium (lacking uracyl + 2% glucose (24h, cultivation) | Shake flask | No. | No. | [41] |
| *A. nidulans* | Integrative | *xlnB* | *A. niger* gox | Maximum level: 2.3 U/mL | *Aspergillus* minimal medium [55]. Xylose was used as inducer (cultivation) | Biore-actor | Yes. The optimum pH of the culture was optimized and the best carbon source searched. Maximum extracellular GOX activity was obtained at pH 5.5 and in the presence of oat spelt xylan as carbon source. | No. | [42] |
| *H. polymorpha* | Episomal | *PMA1* and *MOX* | *S. cerevisiae MFα* | Maximum level: 298 U/gDW | Fed batch using DW minimal medium [56] +2% glucose. Feeding occured using 50% of carbon source. | Biore-actor | Yes. High-density fed-batch cultivations were performed at optimized pH of 5.0 and temperature of 30°C | No. | [7] |
| *K. marxianus* | Episomal | *INU1* | *K. marxianus* *INU1* | 1316 U/gDW | Minimal medium + 2% glucose (24h, preculture); complex medium + 2% sucrose (36h, cultivation) | Shake-flask | No. | Yes. GOX weight: approx. 200 kDa. | This work |
| *K. lactis* | Episomal | *PGK* | *K. lactis* k_l_ killer protein | 174 U/gDW | Minimal medium + 2% glucose (24h, preculture); complex medium + 2% sucrose (36h, cultivation) | Shake-flask | No. | Yes. GOX weight: approx. 300 kDa. | This work |

**^(a)^**NP: not published
